# Managing human-mediated range shifts: understanding spatial, temporal and genetic variation in marine non-native species

**DOI:** 10.1098/rstb.2021.0025

**Published:** 2022-03-14

**Authors:** Luke E. Holman, Shirley Parker-Nance, Mark de Bruyn, Simon Creer, Gary Carvalho, Marc Rius

**Affiliations:** ^1^ School of Ocean and Earth Science, National Oceanography Centre Southampton, University of Southampton, Southampton, UK; ^2^ Zoology Department, Institute for Coastal and Marine Research Nelson Mandela University Ocean Sciences Campus, Gqeberha (Port Elizabeth), South Africa; ^3^ South African Environmental Observation Network (SAEON) Elwandle Coastal Node, Nelson Mandela University Ocean Sciences Campus, Gqeberha (Port Elizabeth), South Africa; ^4^ School of Life and Environmental Sciences, The University of Sydney, Camperdown, Australia; ^5^ Molecular Ecology and Evolution Group, School of Natural Sciences, Bangor University, Bangor, UK; ^6^ Centre for Advanced Studies of Blanes (CEAB, CSIC), Accés a la Cala Sant Francesc 14, 17300 Blanes, Spain; ^7^ Centre for Ecological Genomics and Wildlife Conservation, Department of Zoology, University of Johannesburg, Auckland Park, South Africa

**Keywords:** ascidians, biodiversity, environmental DNA, non-native species, range shifts

## Abstract

The use of molecular tools to manage natural resources is increasingly common. However, DNA-based methods are seldom used to understand the spatial and temporal dynamics of species' range shifts. This is important when managing range shifting species such as non-native species (NNS), which can have negative impacts on biotic communities. Here, we investigated the ascidian NNS *Ciona robusta*, *Clavelina lepadiformis*, *Microcosmus squamiger* and *Styela plicata* using a combined methodological approach. We first conducted non-molecular biodiversity surveys for these NNS along the South African coastline, and compared the results with historical surveys. We detected no consistent change in range size across species, with some displaying range stability and others showing range shifts. We then sequenced a section of cytochrome c oxidase subunit I (COI) from tissue samples and found genetic differences along the coastline but no change over recent times. Finally, we found that environmental DNA metabarcoding data showed broad congruence with both the biodiversity survey and the COI datasets, but failed to capture the complete incidence of all NNS. Overall, we demonstrated how a combined methodological approach can effectively detect spatial and temporal variation in genetic composition and range size, which is key for managing both thriving NNS and threatened species.

This article is part of the theme issue ‘Species’ ranges in the face of changing environments (part I)’.

## 1. Introduction

Biodiversity is undergoing a global redistribution as a result of human influence, with species increasingly found in environments outside their previously reported geographical range [1]. Contemporary climate change is causing species to shift their ranges to accommodate novel environmental conditions [2,3], and human-mediated species introductions dramatically increase the range of non-native species (NNS) [4–6]. This exposes species to abiotic conditions and biotic interactions that are different to those experienced in native habitats. Such changes in distribution can result in a dramatic increase or decrease in population size, or may have a limited detectable immediate effect [1,7]. Understanding these responses is important to answer fundamental ecological and evolutionary questions [8,9], but also for natural resource managers when predicting changes in ecosystem services and natural capital [1,10].

Global biodiversity loss has consistently been shown to reduce ecosystem function and affect the provision of ecosystem services [11,12]. A key driver of biodiversity loss is the introduction of NNS [13], which also imposes a substantial global economic cost [14] and has a dramatic impact on public health [15,16]. In the marine environment, the majority of NNS introductions are associated with transoceanic shipping [5,17,18] and therefore, major ports and harbours are hotspots for NNS. Once a species is introduced to these sites, subsequent (secondary) spread can be facilitated by smaller recreational vessels, marinas and marine infrastructure surrounding these major harbours [19,20]. As the number of introduced NNS is increasing yearly [6,21], improving our understanding of how range shifts of NNS occur through time and space is critical for the design of effective management and mitigation responses.

Natural resource managers have finite budgets and limited information when making decisions simultaneously on a number of NNS with variable or unknown impact [22,23]. For each NNS, managers can attempt to eradicate a population, make efforts to avoid any further expansion into new areas, or acknowledge that control is not possible and work on mitigation strategies [24,25]. These limited options are compounded by the vast costs associated with control or eradication, and even when control methods may be possible, they might be politically or publicly unacceptable [26,27]. Furthermore, control measures can be unsuccessful because of incomplete eradication of the target species or ongoing species reintroductions [22,28,29]. Consequently, managers frequently take no action to control NNS or act only when evidence for both presence and substantial impact has been gathered [30]. It is, therefore, beneficial to develop tools that provide researchers and managers with information to facilitate decision-making. Genetic tools can complement existing methods for assessing NNS range shifts by providing information that would be unfeasible or impossible to produce otherwise [31].

Many disciplines rely on accurate and complete taxonomic information, and this is of particular importance in invasion science [32–34]. Even when NNS can be unambiguously identified, it can be difficult to determine when and where they were first introduced into a region (for example see Hudson *et al.* [35]). Since eradication or control efforts are improved by early detection [36], methods with high sensitivity are needed to increase the likelihood of successful management outcomes. One such method is the isolation of DNA from environmental samples (environmental DNA or eDNA) such as water or sediment for the detection of organisms. Studies have demonstrated that the amplification of DNA barcode regions from eDNA (eDNA metabarcoding) can be used to detect marine NNS [37–40] and that it is a sensitive and accurate method for biomonitoring [41,42]. However, eDNA surveys are rarely used in conjunction with existing methods to detect NNS range shifts, and eDNA metabarcoding can validate, endorse or highlight flaws in current biodiversity management strategies.

During a range expansion, understanding if there was a single NNS introduction event or multiple simultaneous introductions is valuable for managers to target possible source regions, and to effectively manage introduction vectors (for example, ballast waters). As NNS spread across the new region, understanding if expansions are due to local spread or introductions from distant regions is useful to target containment efforts. Finally, after eradication efforts have been conducted, understanding if the reappearance of NNS is due to incomplete eradication or a secondary reintroduction is of value for effective management into the future. The sequencing of DNA isolated from NNS has previously identified the source of an introduction [43,44], provided evidence of multiple introductions [45] and tested if post eradication invasions are a result of incomplete eradiation or reinvasion [46]. Cumulatively, these studies have demonstrated the value of DNA evidence for the management of NNS. Furthermore, observations from both laboratory [47,48] and field studies [49–54] have shown that eDNA can provide population genetics inference, but very little work has used this approach to study NNS [55].

Here, we combined eDNA metabarcoding, mitochondrial gene sequencing and non-molecular biodiversity surveys to study four NNS that are directly relevant to marine natural resource managers. First, we evaluated if the NNS shifted their ranges over decadal time scales and compared each range shift to historical data. Secondly, we evaluated changes in genetic diversity and haplotype composition for each NNS between two sampling occasions across the sampled coastline. Finally, we examined how spatial genetic variation data can inform the management of range shifting species by comparing eDNA metabarcoding data to biodiversity survey and mitochondrial DNA sequence datasets.

## 2. Methods

### (a) Fieldwork and historical biodiversity data

**Figure 1.**
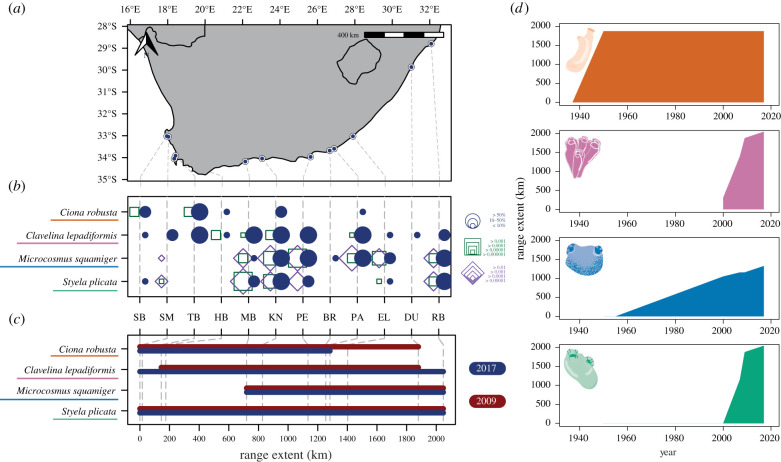
(*a*) Map depicting the coastline of South Africa; sampling sites are shown as blue points, full details in electronic supplementary material, table S1. (*b*) Bubble plot showing the incidence of four non-native ascidians across the sampling sites shown in the map from west to east. Blue bubbles show the percentage cover recorded from rapid assessment surveys and square outlines show the results of eDNA metabarcoding surveys conducted concurrently. Results from COI are shown with green squares and 18S shown with purple squares, the size of each point or square shows the comparative density measured by relative read abundance per sample. Site codes correspond with sites as detailed in electronic supplementary material, table S1. (*c*) Line plot showing range extent over the surveyed coast for 2009 (dark red) rapid assessment surveys from Rius *et al.* [57] and surveys conducted in 2017 presented here (blue). The location of each site across the coastline is shown with grey dashed lines. (*d*) Historical maximum range extent for each of the featured species across the coastline of South Africa; *y*-axis is kilometres of extent, *x*-axis is year, colour indicates each of the species indicated according to labels in (*b*) and (*c*).

The coastline of South Africa is an ideal system to study range shifting species and their management. South Africa has been subject to intense human impact and many species invasions have been documented across the three environmentally varied coastal ecoregions [56–58]. Moreover, data from rapid assessment surveys (a non-molecular biodiversity survey technique) [59] have been previously collected and mitochondrial sequence data have been generated for NNS along the entire coastline [57]. Furthermore, historical data are available for a range of relevant species [60–62] providing an insightful opportunity to conduct a spatial and temporal analysis of range expansions. Here, we selected 12 human-impacted sites and conducted surveys (see details below) between October and November 2017. The sampled sites were the 11 sites previously sampled in 2007 and 2009 by Rius *et al.* [57], which included all major harbours and a number of marinas, and a new marina constructed post 2009 (figure 1*a*, with full details in electronic supplementary material, table S1). Collectively, the sites encompass the main introduction points for marine NNS into the South African coastline.

At each sampling site, a rapid assessment survey was conducted following Rius *et al.* [57], targeting non-native ascidian species (Class: Ascidiacea). Ascidians are unique species for studying range expansions as they are successful invaders [63] and have a relatively short pelagic larval phase, meaning that long-distance dispersal can only be achieved through anthropogenic transport of species [64]. For each site, species abundance was ranked as absent (0%), scarce (less than 10%), common (10–50%) or dominant (greater than 50%) based on observations of substrate coverage as in Rius *et al.* [57].

Rapid assessment survey data from 2007 to 2009 were sourced from Rius *et al.* [57] for the species of interest. Additionally, historical incidence data were extracted from several taxonomic publications [60–62,65,66]. These investigations are not an exhaustive survey of the coastline, but they provide valuable historical species incidence data over the last century and are therefore of value in gaining a broad understanding of range shifts over time.

### (b) Sample collection, DNA extraction and Sanger sequencing

Tissue samples were collected from the field in 2017 for species for which genetic data were available from the 2009 surveys (*Ciona robusta*, *Clavelina lepadiformis*, *Microcosmus squamiger* and *Styela plicata*). These species can be morphologically identified in the field (but see the recent taxonomic delineation of cryptic species within the genus *Ciona* [32]). Samples were collected where sufficient numbers of individuals per species were present at a site to provide a reasonable estimate of genetic diversity (minimum 10 individuals), with 30+ individuals per species being the target at each sampling site. Organisms were sampled by hand, with no adjacent (within 0.3 m) individuals collected, and dissected within 6 h (see details of research permit in the Acknowledgements). For each sampled individual, approximately 10 mm^2^ of tissue from around the siphons was dissected using tools decontaminated with 10% bleach solution (3.5% chlorine), except in the case of *C. lepadiformis* for which a single zooid was removed from the tunic and stored. Tissue samples were preserved in 100% ethanol and stored at ambient temperature during transportation, and then stored at −80°C in the laboratory until later DNA extraction.

DNA from ascidian tissue samples was extracted using the Qiagen (Hilden, Germany) DNeasy Blood and Tissue Kit (96 Well Format) following the manufacturer's recommended protocol with one blank control per extraction run. A section of the cytochrome c oxidase subunit I gene (COI) was sequenced for all tissue samples aiming to cover the entire section previously analysed in Rius *et al.* [57]. Each PCR contained 6 µl of Applied Biosystems (Foster City, CA, USA) AmpliTaq GOLD 360 Mastermix, 1.8 µl of oligonucleotide mix (5 µm concentration per primer), 1.2 µl of undiluted template DNA and PCR quality water up to 12 µl total reaction volume. The reaction conditions varied by primer set and are listed in the electronic supplementary material, table S2a. During preliminary trials a set of primers were designed and validated for *M. squamiger* (sequence details in electronic supplementary material, table S2b); existing primer sets [33,67,68] were optimized for the remaining three species. Successful amplification was confirmed using gel electrophoresis and PCR products were cleaned using Applied Biosystems ExoSAP-IT Express following the manufacturer's recommended protocol. Cleaned products were normalized to approximately 50 ng µl^−1^ and 5 µl of sample was added to each 5 µl of the forward or reverse primers (5 µm) used in the initial PCR. These samples were sent for sequencing using the Macrogen Europe (Amsterdam, The Netherlands) EZ-Seq service. Resultant chromatogram files were analysed using Geneious Prime (v. 2020.2.4) (Biomatters Ltd, Auckland, New Zealand). For each sequence the forward and reverse traces were aligned and sequences with ambiguities or failed reactions were re-sequenced from the initial PCR once and subsequently discarded if poor results persisted. The 764 COI sequences from Rius *et al.* [57] were added to the analysis and trimmed, truncated and aligned with the experimental data as follows. For each species, sequences were trimmed to remove primer binding and poor-quality regions and aligned using the Geneious Alignment Tool. Subsequently, each alignment was manually checked to confirm complete alignment, and short sequences that did not overlap at all polymorphic regions or had ambiguous base calls were discarded.

### (c) Environmental DNA metabarcoding

Before each rapid assessment survey, surface seawater was sampled from the top 10 cm for eDNA metabarcoding following Holman *et al.* [69]. Briefly, three replicate 400 ml water samples were filtered on site with a 0.22 µm polyethersulfone enclosed filter. Filters were preserved with Longmire's solution until DNA extraction following Spens *et al.* [70]. Data generated from these samples is presented in Holman *et al.* [69] with the aim of conservatively characterizing whole community diversity. COI and ribosomal RNA (18S) data targeting metazoans [71,72] were reanalysed as follows for accurate ascidian species detection. Primer regions were removed from forward and reverse reads using the default settings of Cutadapt (v. 2.3) [73]. Sequences were denoised and an ASV (amplicon sequence variant) by sample table generated using DADA2 (v. 1.12) [74] in R (v. 3.6.1) [75] with parameters as in Holman *et al.* [69]. Recent work has highlighted that different bioinformatic methods have an effect on the resolution of intraspecific variation of eDNA metabarcoding data [47,50,76]. Therefore, in addition to the sequenced tissue samples and DADA2 methods outlined above, we reanalysed the COI data using the *unoise3* algorithm (hereafter UNOISE3) [77] as follows. Raw COI paired-end fastq data from Holman *et al.* [69] was merged using usearch (v. 11.0.667) [77] with the following parameters *-fastq_maxdiffs 15 -fastq_pctid 80*. Primer sequences were then stripped from each merged read using Cutadapt (v. 3.1) [73] under the default parameters, and reads longer than 323 and shorter than 303 base pairs (±10 from the expected size of 313) were discarded. Reads from all samples were pooled, and singletons and reads with an expected error greater than 1 were discarded using vsearch (v. 2.15.1) [78]. The *unoise3* algorithm from usearch was then used to generate ASVs with *-unoise_alpha* set at 5 as recommended for resolving metazoan intraspecific variation with a COI fragment of 313 base pairs in length [50]. Sequences were then mapped back to the ASVs using the -u*search_global* function of vsearch with an *-id* parameter of 0.995 to produce an ASV by sample table.

To provide an initial taxonomic assignment all ASVs were compared using a BLAST (v. 2.6.0+) search with no limits on sequence similarity or match length to the NCBI *nt* database (downloaded 16 May 2019). Reference sequences for COI and 18S are publicly available for all four target species in this database. Taxonomic assignments were then parsed using a custom R function (*ParseTaxonomy*, doi:10.5281/zenodo.4671710) with the default settings. The taxonomic assignments were subset to include only those with a hit to species in the class Ascidiacea*.* The following quality control steps were then applied to each dataset. The data were filtered to only retain ASVs that appeared in more than one replicate sample. For any ASVs detected in both the negative and experimental control samples, the maximum number of reads in the negative controls were subtracted from the experimental control samples. Reads were then divided by the total number of reads per sample and relative proportions were used in all subsequent analyses; technical replicates per site were averaged. The remaining ASVs were then taxonomically checked manually using the online National Centre for Biotechnology nucleotide BLAST search function against the *nt* databases (last accessed on 1 October 2020) under default megablast parameters. For each ASV in the COI dataset, taxonomy was only assigned at species level if multiple, independent sequences had a match greater than 97% identity (with 100% coverage) with no other species within 97% of the target ASV. For the 18S dataset, a 100% match (with 100% coverage) between the subject ASV and database sequences was required for taxonomic confirmation. Additionally, as some taxa within the same genera have near 100% similarity at the 18S region, taxonomy was only assigned to species if organisms from the same genera were in the database with at least 1 base pair between the query and species from the same genera. Following taxonomic annotation, ASVs assigned to the same species were merged for the distribution datasets. ASVs were kept separate for the haplotype reconstruction of the COI data.

### (d) Data manipulation and statistical analyses

Distances between sites along the coast were estimated by drawing a transect 1 km parallel to the coastline in Google Earth Pro (v. 7.3.2.5776) and calculating the distance between each pair of sites. The study area was plotted using the function *map* from the package *maps* (v. 3.3.0). Sequenced COI regions from 2009 to 2017 were aligned separately for each species using the Geneious aligner in Geneious Prime; alignments were truncated to include only overlapping regions. Sequences were manipulated using the *SeqinR* package in R (v. 4.2-5) [79]. Nucleotide and haplotypic diversity were calculated using the *nuc.div* and *hap.div* functions from the *pegas* package (v. 0.14) [80]. For each species, an alignment was created between the tissue sampled COI sequences and the eDNA metabarcoding derived haplotypes. The region of overlap was extracted and used in subsequent analyses. Haplotype frequencies were calculated per site for the tissue-derived sequences and the different bioinformatic analyses of eDNA metabarcoding data. Minimum spanning network haplotype maps [81] were created using the default settings of PopArt (v. 1.7) [82]. Analyses of molecular variance (AMOVA) were performed using the function *poppr.amova* from the *poppr* package (v. 2.8.6) [83]. AMOVA models were structured to analyse the effect of sampling year and sites for each species. All data analyses were conducted in R (v. 4.0.3) unless otherwise stated.

## 3. Results

### (a) Range shifts

Rapid assessment surveys found that non-native ascidians known to be broadly restricted to warmer waters (*M. squamiger* and *S. plicata*) [57] showed distributions principally limited to the southern and eastern coastlines (figure 1*b*). By contrast *C. robusta* and *C. lepadiformis* were found along most of the coastline. We found no change across years in range extent for *M. squamiger* and *S. plicata*, a decrease in easternly range for *C. robusta* and an expansion of range both westerly and easterly for *C. lepadiformis* (figure 1*c*)*.* Historical total range extent data (figure 1*d*) showed more recent increases in range for *C. lepadiformis* and *S. plicata* compared to *C. robusta* and *M. squamiger*. The COI and 18S eDNA metabarcoding data showed mixed results. There was good agreement between detections from eDNA and rapid assessment surveys in *M. squamiger* and *S. plicata* (figure 1*b*). However, 18S entirely failed to detect *C. robusta* or *C. lepadiformis*, and COI demonstrated a number of false-negative metabarcoding detections in these species (figure 1*b*). For sites sharing detections from eDNA metabarcoding and rapid assessment surveys, eDNA metabarcoding data and field density estimates showed a non-significant relationship (18S *p* = 0.052, COI *p* = 0.297) (see electronic supplementary material, note 1 for details).

### (b) Changes in genetic composition

**Figure 2.**
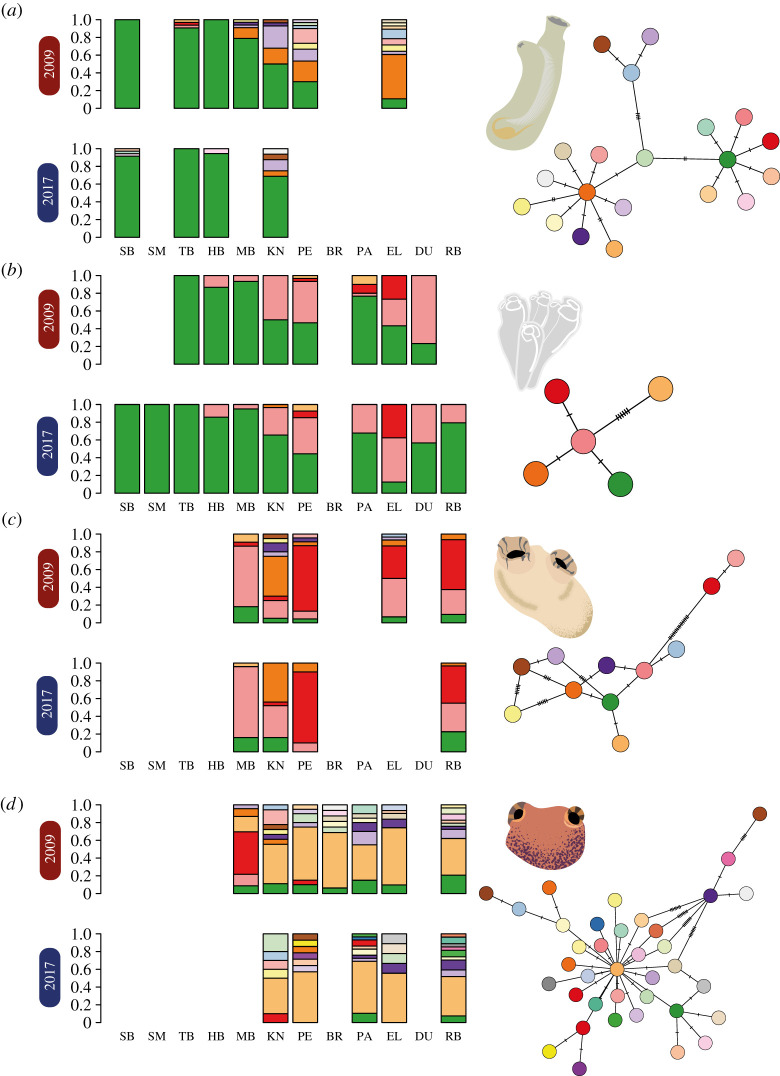
Mitochondrial DNA COI haplotype proportions for (*a*) *Ciona robusta*, (*b*) *Clavelina lepadiformis*, (*c*) *Styela plicata* and (*d*) *Microcosmus squamiger* along the South African coastline. Results are shown for surveys conducted in 2009 and 2017 for each species; site abbreviations follow electronic supplementary material, table S1. Haplotype networks based on minimum spanning distance are shown for each species with colours matching the bar plot within species; the number of cross-hatches indicates the mutation steps between haplotypes.

A total of 1320 sequencing reactions generated 660 bi-directionally sequenced COI sequences. After alignment and quality control, 541 samples remained with complete alignment and no missing site information, 88 for *C. robusta*, 261 for *C. lepadiformis*, 90 for *M. squamiger* and 102 for *S. plicata*. After combining the COI sequences with previously sequenced samples from 2009 [57], alignments were 626, 440, 635 and 599 base pairs in length for *C. robusta*, *C. lepadiformis*, *M. squamiger* and *S. plicata*, respectively. Observed haplotype richness across both sampling years and all sites was highest in *M. squamiger* followed by *C. robusta*, *S. plicata* and *C. lepadiformis* (figure 2). There was no statistically significant difference between nucleotide or haplotype diversity between sampling years across all species (*p* > 0.05 in all cases, see electronic supplementary material, note 2, for full model output and details). Additionally, AMOVA models found no significant differences between sampling years across all species (*p* > 0.05 in all cases, see electronic supplementary material, table S3, for full model outputs), but significant differences between sampling sites within years (*p* < 0.05 in all species, see electronic supplementary material, table S3, for full model outputs). In all species, the greatest proportion of the genetic variance was found between samples, then within sampling sites, followed by the variance between sampling sites (electronic supplementary material, table S3). As shown in figure 2, haplotype frequencies agreed with the AMOVA analyses, showing stable patterns of genetic variation occurring between years and variation in haplotype frequencies across the study system (figure 2).

**Figure 3.**
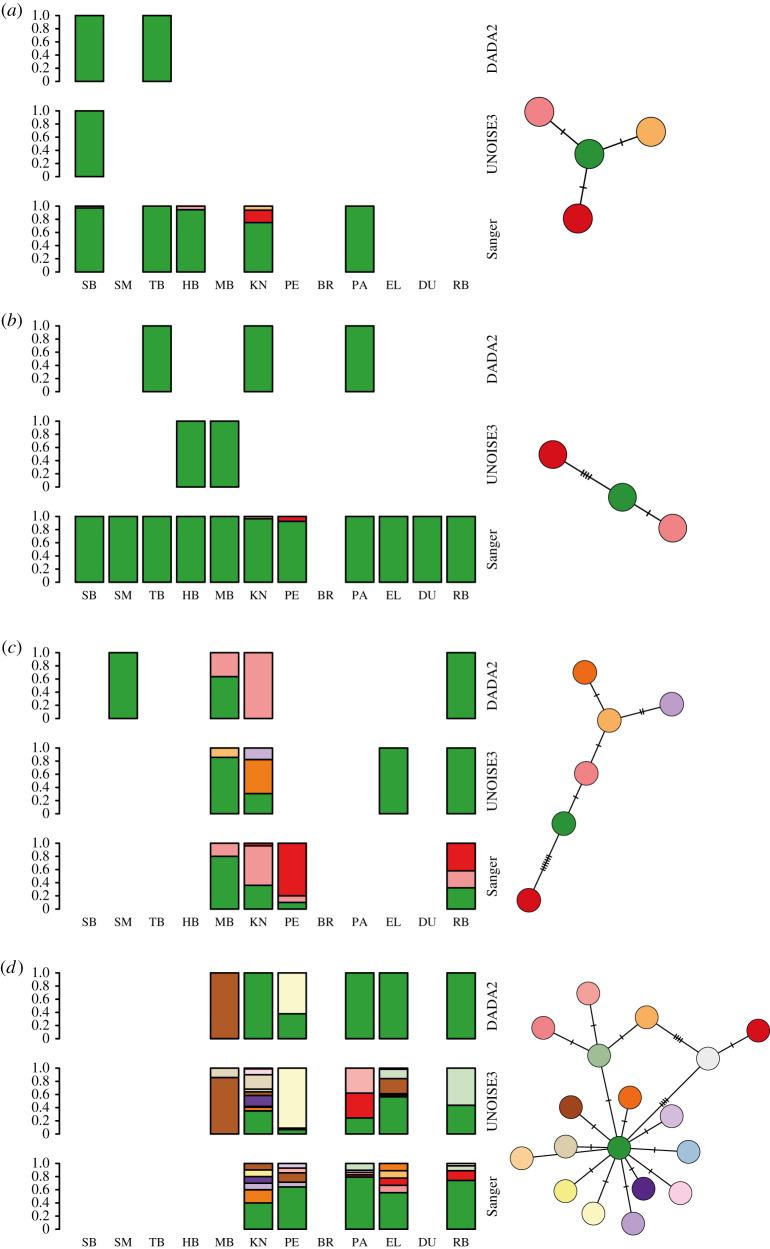
Haplotype proportions recovered using eDNA metabarcoding for (*a*) *Ciona robusta*, (*b*) *Clavelina lepadiformis*, (*c*) *Styela plicata* and (*d*) *Microcosmus squamiger* along the South African coastline. Results are shown for analysis of COI eDNA metabarcoding data using the denoising software DADA2 and UNOISE3 for each species; site abbreviations follow electronic supplementary material, table S1. Haplotype networks based on minimum spanning distance are shown for each species with colours matching the bar plot within species; the number of cross-hatches indicates single nucleotide mutation steps between haplotypes.

After aligning the shorter sequences derived from eDNA metabarcoding data to the sequenced COI region, alignments were 191, 258, 289 and 286 base pairs in length for *C. robusta*, *C. lepadiformis*, *M. squamiger* and *S. plicata*, respectively. Regardless of bioinformatic method and across species, the eDNA metabarcoding data did not recover all the haplotype sequences derived from tissue (figure 3). For *C. robusta* and *C. lepadiformis*, only the most common haplotype from tissue-derived sequences was recovered across both bioinformatic methods from the eDNA data. In the case of *S. plicata*, DADA2 recovered two haplotypes from the eDNA data also found in the tissue-derived sequences, while UNOISE3 only found a single sequence in common. However, UNOISE3 detected three haplotypes unseen in the other datasets. Finally, for *M. squamiger*, DADA2 and UNOISE3 recovered two and eight haplotypes shared with the tissue-derived sequences, respectively. DADA2 recovered one haplotype unique to the eDNA data while UNOISE3 recovered four.

## 4. Discussion

Here, we found both losses and gains in range size across sampling years for four non-native ascidian species, with no consistent pattern emerging when introduction dates were compared. For all species, we found substantial haplotype variability across the study region but no significant change in genetic variation for almost a decade. Finally, eDNA metabarcoding data recovered broad NNS incidence trends and for some species was as accurate as non-molecular surveys. Most dominant haplotypes from tissue samples were detected with eDNA metabarcoding but fine-scale genetic patterns could not be resolved using the eDNA metabarcoding data. Cumulatively, the evidence demonstrates that both DNA and non-DNA biodiversity survey methods can be used in combination to evaluate the role of genetic variation on range shifts and to inform natural resource managers.

Non-DNA biodiversity surveys found that *C. lepadiformis* expanded its range by 168.4 km since surveys in 2009, for an assumed rate of 21.1 km per year. This is in line with previous studies that found an average marine non-native spread rate of 44.3 km per year [84], with values of 16 km per year for tunicates, 30.0 km per year for barnacles and 20 km per year for a bryozoan species [85]. By contrast, we observed a range contraction for *C. robusta* (figure 1), which was unexpected as there are few studies showing range contraction in the introduced range for marine species. However, previous work has identified biotic resistance for invasions of several species in the genus *Ciona* [9,86], and so it might be feasible for local species to have begun predating on *C. robusta* during the 80+ years it has been documented in South Africa (figure 1*d*). A lack of any western increase in range for *M. squamiger* might be explained by the species inability to mature to reproductive age in the colder sea temperature on the western coast [57]. Further range expansions or contractions (eastwards for *M. squamiger* and east or westward for *S. plicata*) cannot be ruled out as observations of these species extended to the margins of the sampled area. It is important to note that the harbours and marinas in this study act as islands of suitable habitat, and the frequency of introductions outside these areas is relatively uncertain. Further surveys of surrounding hard benthic environments are required to understand the role of artificial environments across the coastal ecosystem. Overall, these patterns demonstrate that the spread of marine NNS is not characterized by a continuous expansion of range, but rather by a complex picture of expansions and contractions in response to dynamic abiotic and biotic conditions.

A consistent pattern of genetic differentiation emerged across the studied species, with significant differences across sampling sites and persistence of similar haplotypes across time (figure 2). Previous studies of temporal changes in the genetic diversity of non-native ascidians have found some evidence for genetic differences over time [87,88]. By contrast other work has found relatively stable genetic diversity over several years [89,90]. In our study, the time between sampling occasions (i.e. 2009 and 2017) represents between four and 24 generations, depending on the species [91–93]. Therefore, dramatic changes in haplotype frequencies could only be as a result of the anthropogenic transfer of haplotypes between sites or changes in site frequencies in response to high mortality events (for example, extreme weather events). These types of changes have been documented in ascidian species elsewhere [8,88,94], and a large number of NNS introductions have been documented in South African marinas and harbours supporting the regional transfer of these organisms [57,58]. It is, therefore, somewhat surprising that across four different species, all of which are known to be transported anthropogenically, there was little evidence of shifts in haplotype composition. Consequently, our results demonstrated that the studied NNS are well-established and are not subject to high levels of mortality or genetic bottlenecks that may affect population viability. It may be that these well-established haplotypes prevent newcomers from successfully inhabiting the site, which would explain our observations of persistent haplotype composition across the studied time period.

We found that eDNA metabarcoding captured similar incidence data as rapid assessment surveys for some species, and performed poorly for others. Previous work has identified that NNS can be detected using eDNA metabarcoding [37,39], but these surveys aimed at detecting any NNS rather than a specific set of target taxa. Several studies have identified that general target metabarcoding primers show lower reliability and sensitivity compared to species-specific quantitative PCR assays [95,96]. Additionally, previous work has identified that in some cases different bioinformatic methods carry variable sensitivity [97], although this effect is fairly minimal in this dataset (see electronic supplementary material, note 3). There is also some evidence that increased sensitivity may be possible with greater sequencing depth offered by newer sequencing technologies [98]. Indeed, here we found that the total proportion of reads per sample for each target ascidian was low (figure 1*b* and electronic supplementary material, table S1 of Holman *et al.* [69]), which may have contributed to some of the false-negative detections. Managers should, therefore, be aware that general metabarcoding primers will perform well for the detection of some important NNS but others may be missed due to poor sensitivity. In cases when a list of priority species can be assembled, mixed DNA positive control samples or trials with aquaria of known composition (for example, Holman *et al.* [99]) would provide information on which NNS might be overlooked by eDNA metabarcoding. Experimental trials are important as *in silico* approaches to evaluate primer bias do not always correspond with experimental results, as shown here by non-detection of known species despite no primer mismatches in the 18S dataset (see electronic supplementary material, note 4). Inevitably, there will be a cost-benefit trade-off between using imperfect broad metabarcoding assays for monitoring unknown invaders, and expending resources on the development and application of eDNA tools targeting specific known NNS.

In some cases, natural resource managers might be interested in tracking invasions using haplotype data [100]. Here, we showed that eDNA metabarcoding with broad-target primers resolves broad-scale patterns of haplotype diversity (figure 3). However, fine-scale genetic variation was not recovered in our study, indicating that targeted eDNA amplicon sequencing [53] might be more appropriate when this level of genetic data is required. As with biodiversity incidence data, the management objectives for a given NNS determine how haplotype sequencing should be implemented. If large numbers of tissue samples can be easily collected and there are sufficient resources, then sequencing the tissue directly might be more appropriate. By contrast, if the aim is a broad-scale analysis across a large or difficult-to-sample area, resolving haplotype data from eDNA metabarcoding data might be preferable. Overall, eDNA-based techniques show great potential for NNS detection, but for our target taxa, we demonstrated that current biodiversity surveys and direct tissue sequencing are more reliable for the detection of NNS and genetic composition. It is important to note that there are several key advantages of eDNA-based methods compared to the other tools used in this work. Firstly, eDNA samples can be collected with minimal training and the sequenced DNA provides an unambiguous identification, provided reference data are available [38,39]. Secondly, eDNA-based methods can be automated and can scale to a much greater survey effort at reduced cost compared to other methods [101]. Finally, the limitations described above concerning the sensitivity of eDNA-based incidence data and lack of resolution of eDNA-based haplotype data can be attributed to the use of metabarcoding with broad-target primers. Reanalysing the samples with metabarcoding primers for more specific groups or using species-specific qPCR assays [96] would provide increased sensitivity and accuracy.

Overall we demonstrated how our combined methodological approach can effectively detect spatial and temporal trends of range shifts and genetic differentiation, but also monitor biodiversity changes of both threatened and NNS. The strengths of eDNA or DNA-based biomonitoring demonstrated here for the detection of range shifting species make them a pragmatic choice for natural resources managers. These tools can provide managers with additional sensitivity and accuracy when monitoring biodiversity in human-impacted environments.

## Data accessibility

Raw and processed data are available online via Zenodo: https://doi.org/10.5281/zenodo.5046378. Raw sequence data associated with analyses can be found under European Nucleotide Archive Project Accession PRJEB38452. Haplotype sequences have the following NCBI GenBank accession numbers, *Ciona robusta*: MZ882298–MZ882305, *Clavelina lepadiformis*: MZ882307–MZ882311, *Microcosmus squamiger*: MZ882313–MZ882337, *Styela plicata*: MZ882344–MZ882348. Associated R scripts and intermediate files are available online via Zenodo: https://doi.org/10.5281/zenodo.5046378.
