# Correction to: ‘Managing human-mediatedrange shifts: understanding spatial,temporal and genetic variation in marinenon-native species’ (2022) by Holman *et al*.

**DOI:** 10.1098/rstb.2022.0284

**Published:** 2022-11-07

**Authors:** Luke E. Holman, Shirley Parker-Nance, Mark de Bruyn, Simon Creer, Gary Carvalho, Marc Rius

*Phil. Trans. R. Soc. B* **377**, 20210025. (Published online 24 January 2022) (https://doi.org/10.1098/rstb.2021.0025)

After publication, the authors identified errors in two panels of figure 1.

**Figure:**
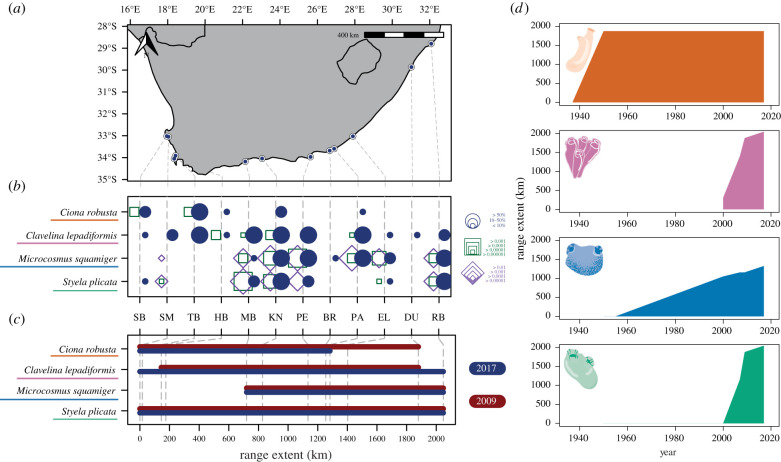

In panel (*b*), the legend for the relative read abundances of COI and 18S markers previously showed values for the incorrect marker, with the values shown for COI (green) showing those for 18S (purple) and vice versa.

Owing to an error in plotting, the historic range of *Clavelina lepadiformis* (panel (*d*)) showed a gradual increase from 1950 to 2000; however, the species was first recorded in 2000. Additionally, the plots for *Microcosmus squamiger* and *Styela plicata* were incorrectly switched.

None of the above errors affected the conclusions or analyses in the study.

This has been corrected on the publisher's website.

